# The impact of radiation-induced temporal lobe injury on cognition and structure across the whole brain in nasopharyngeal carcinoma patients

**DOI:** 10.1016/j.ibneur.2026.03.006

**Published:** 2026-03-12

**Authors:** Peng Xie, Siwen Liu, Jingjing Han, Pengwei Yan, Jianfeng Wu, Yizhi Ge, Yesong Guo

**Affiliations:** a Department of Radiation Oncology, The Affiliated Cancer Hospital of Nanjing Medical University, Jiangsu Cancer Hospital, Jiangsu Institute of Cancer Research, Nanjing 210009, China; b Department of Oncology, The Affiliated Cancer Hospital of Nanjing Medical University, Jiangsu Cancer Hospital, Jiangsu Institute of Cancer Research, Nanjing 210009, China; c Department of Radiotherapy, The Affiliated Cancer Hospital of Nanjing Medical University, Jiangsu Cancer Hospital, Jiangsu Institute of Cancer Research, Nanjing 210009, China

**Keywords:** Nasopharyngeal carcinoma, Radiotherapy, Radiation-induced temporal lobe injury, Cognitive impairment, Structural magnetic resonance imaging, Voxel-based morphometry

## Abstract

**Introduction:**

Radiation-induced temporal lobe injury (RI-TLI) is one of the most common late-stage complications after radiotherapy for nasopharyngeal carcinoma (NPC), which seriously affects patients’ cognitive function and quality of life. However, the mechanism by which RI-TLI affects the gray matter (GM) and white matter (WM) across the whole brain, leading to cognitive impairment, is currently unclear in NPC patients.

**Methods:**

One year after the end of radiotherapy, 32 NPC patients with RI-TLI (RI-TLI group) and 38 NPC patients without RI-TLI (nRI-TLI group) were included in this study. The Montreal Cognitive Assessment (MoCA) was applied to evaluate the differences of cognitive function between RI-TLI and nRI-TLI groups. In addition, T1 structural magnetic resonance imaging data were acquired, and then the whole-brain voxel-based morphometry was employed to compare the differences of GM and WM between groups. The relationships between GM and WM of abnormal brain regions and MoCA scores were explored. Finally, receiver operating characteristic (ROC) curve was performed to determine the suitability of altered brain structure for distinguishing RI-TLI from nRI-TLI.

**Results:**

Compared with nRI-TLI group, RI-TLI group exhibited decreased attention, delayed recall scores and total scores of MoCA. In addition, RI-TLI group exhibited decreased GM volume in the right middle temporal gyrus, left hippocampus, right superior temporal gyrus, right superior frontal gyrus and decreased GM density in the left superior frontal gyrus, right superior frontal gyrus, right supplementary motor area. Moreover, RI-TLI group demonstrated decreased WM volume in the right inferior temporal gyrus, left hippocampus, right superior temporal gyrus, right medial superior frontal gyrus and decreased WM density in the left superior frontal gyrus, right superior frontal gyrus. The attention scores of MoCA were positively associated with GM volume of the right superior temporal gyrus, right superior frontal gyrus, GM density of the left superior frontal gyrus, right superior frontal gyrus, WM density of the left superior frontal gyrus. The delayed recall scores of MoCA were positively related to GM volume of the left hippocampus, WM density of the left superior frontal gyrus. The total scores of MoCA were positively associated with GM volume of the left hippocampus, WM volume of the left hippocampus, right superior temporal gyrus, WM density of the left superior frontal gyrus. ROC analysis demonstrated that altered GM and WM might be helpful for distinguishing RI-TLI from nRI-TLI.

**Conclusion:**

Compared to nRI-TLI patients, RI-TLI patients exhibit more severe cognitive impairment alongside decreased GM and WM in both the temporal lobe and prefrontal regions. The observed structural alterations are associated with the severity of cognitive deficits and may serve as potential neuroimaging markers for RI-TLI.

## 1 Introduction

Nasopharyngeal carcinoma (NPC) is one of the most common head and neck malignancies (Chua et al., 2016). Every year there are about 80000 new cases of NPC in the world, which is very common among Asians (Chang et al., 2021). The total incidence rate in Asia is as high as 85% (Wei et al., 2017). In view of the anatomical characteristics of NPC and its sensitivity to radiation, simple radiotherapy or simultaneous radiotherapy and chemotherapy are the key treatment methods for NPC (Tian et al., 2019). With the development of radiotherapy technology, the precision radiotherapy can more accurately control the irradiation of tumors, while also limiting the irradiation dose for organs at risk and reducing damage to surrounding normal tissues. This not only increases the efficacy but also reduces the occurrence of subsequent complications when compared with traditional radiotherapy (Co et al., 2016, Mao et al., 2016, Kong et al., 2018). However, NPC radiotherapy still brings many late complications to patients, such as dry mouth, swallowing difficulties, radiation-related oral mucositis, radiation related dermatitis, radiation-induced temporal lobe injury (RI-TLI), radiation-related osteomyelitis, muscle fibrosis, cognitive dysfunction, etc (Liao et al., 2019, Bian et al., 2015, Gérard et al., 2017, Turnquist et al., 2020, Zhou et al., 2020a, Lam et al., 2012). These late-stage injuries are usually irreversible and lack effective treatment methods. Among them, RI-TLI is one of the most serious late-stage complications of NPC (Zhou et al., 2020a, Zheng et al., 2022, Wu and Tam, 2020, Hou et al., 2022, Pan et al., 2024). Studies have shown that the median latency of RI-TLI is 33 months, which can be asymptomatic or lead to a series of neurological complications such as cognitive decline, memory loss, and epileptic seizures, affecting the quality of life of patients and even endangering their lives (Zeng et al., 2014). Therefore, it is crucial to reduce the occurrence of RI-TLI after radiotherapy for NPC patients.

Radiation-related cognitive dysfunction is one of the common complications of NPC radiotherapy, often occurring more than 6 months after radiotherapy (Kiang et al., 2016). It is currently believed that the occurrence of radiation-induced cognitive dysfunction in NPC is related to the abnormal functional connectivity of brain networks centered around the bilateral hippocampus and temporal lobe (Chen et al., 2017). The extensive neural fiber connections between the temporal lobe and other regions in the brain may be the basis for their involvement in cognitive function. Therefore, RI-TLI is highly likely to play an important role in the pathogenesis of cognitive impairment in NPC patients after radiotherapy. Differences of brain activities were identified within the radiation field (such as bilateral temporal lobes) and outside (such as bilateral insular cortex and left frontal lobe) in NPC patients (Zhang et al., 2021). These findings proposed that radiation-induced brain injury may be a multi system lesion involving both temporal lobe and other regions in the brain (Zhang et al., 2021). In addition, it was found that brain activity in the bilateral superior temporal gyrus and left insula had an accuracy of over 80% in identifying the potential development of radiation-induced encephalopathy, revealing that brain activity can serve as a sensitive imaging biomarker for predicting radiation-induced encephalopathy (Zhang et al., 2021). Abnormal brain network connections were found in late-stage patients after radiotherapy, which were significantly correlated with the Montreal Cognitive Assessment (MoCA) scores (Ma et al., 2016). In addition, the connectivity of the hippocampus was correlated with attention scores in MoCA (Ma et al., 2016).

The most common neurotoxic effect of cranial radiation is not focal necrosis, but diffuse brain injury (Duan et al., 2016, Mitchell et al., 2020). Radiation induced brain damage is not limited to specific brain region and can also induce abnormalities in brain regions other than the temporal lobe. The brain functional connectivity of NPC patients at different stages after radiotherapy decreased to varying degrees (Leng et al., 2021). Most patients within one month and more than one year after radiotherapy had significantly reduced functional connectivity in the insula, and most brain regions such as the frontal and parietal lobes in each group of patients after six months or more after radiotherapy had significantly reduced functional connectivity (Leng et al., 2021). It is speculated that brain functional connectivity is in a dynamic state of injury and reconstruction, and the ability to reconstruct gradually weakens with time delay after radiotherapy (Leng et al., 2021). Interestingly, patients at all stages showed a significant decrease in functional connectivity in the middle temporal gyrus, suggesting that the temporal lobe damage in NPC patients after radiotherapy is more fixed, and the damage in this brain region may be related to memory, cognition, and executive function (Leng et al., 2021). These findings have focused on the functional connectivity changes in the temporal lobe after radiotherapy, however, whether other brain regions of the brain network will exhibit abnormalities were unclear.

The research on neurocognition after radiotherapy mainly focuses on two aspects: brain structure and brain function. However, previous study has found that there are changes in brain functional connections before the abnormality of brain structure occurs (Ren et al., 2019). Conducting research on brain function can help reveal potential biomarkers of brain injury, and the functional connectivity has plasticity, which may provide valuable training for further functional recovery treatments (Kelly and Castellanos, 2014, Luft et al., 2014). However, the changes in brain structure caused by radiation are more stable than functional changes. The strength of functional connectivity between multiple brain regions and the hippocampus decreased after radiotherapy, and the plasticity of hippocampal network connectivity strength was dose-dependent (Chen et al., 2017). Low dose radiation damage can cause transient neurological damage within 1–3 months, leading to mild or recoverable cognitive impairment, but high doses (>30 Gy) can cause permanent damage (Chen et al., 2017). Early monitoring of neural network functional connectivity strength and guiding radiotherapy dosage can ensure the repair of cognitive function (Chen et al., 2017). However, the strength and dose distribution of neural network functional connections between regions change over time, which may lead to varying degrees of cognitive decline, thus requiring further structural magnetic resonance imaging (MRI) research (Chen et al., 2017).

Therefore, this study aimed to explore the differences of cognitive function and brain structure including GM and WM between NPC patients with RI-TLI (RI-TLI group) and NPC patients without RI-TLI (nRI-TLI group) one year after the end of radiotherapy. We hypothesized that the RI-TLI group would demonstrate more severe cognitive impairment and decreased GM/WM volumes/densities in both temporal and extra-temporal regions compared to the nRI-TLI group. Furthermore, we aimed to explore whether the severity of cognitive impairment correlates with the extent of GM/WM alterations in these brain regions. We also investigated the potential of these structural measures to differentiate between patients with and without RI-TLI.

## 2 Materials and methods

### 2.1 Participants

**Fig. 1:**
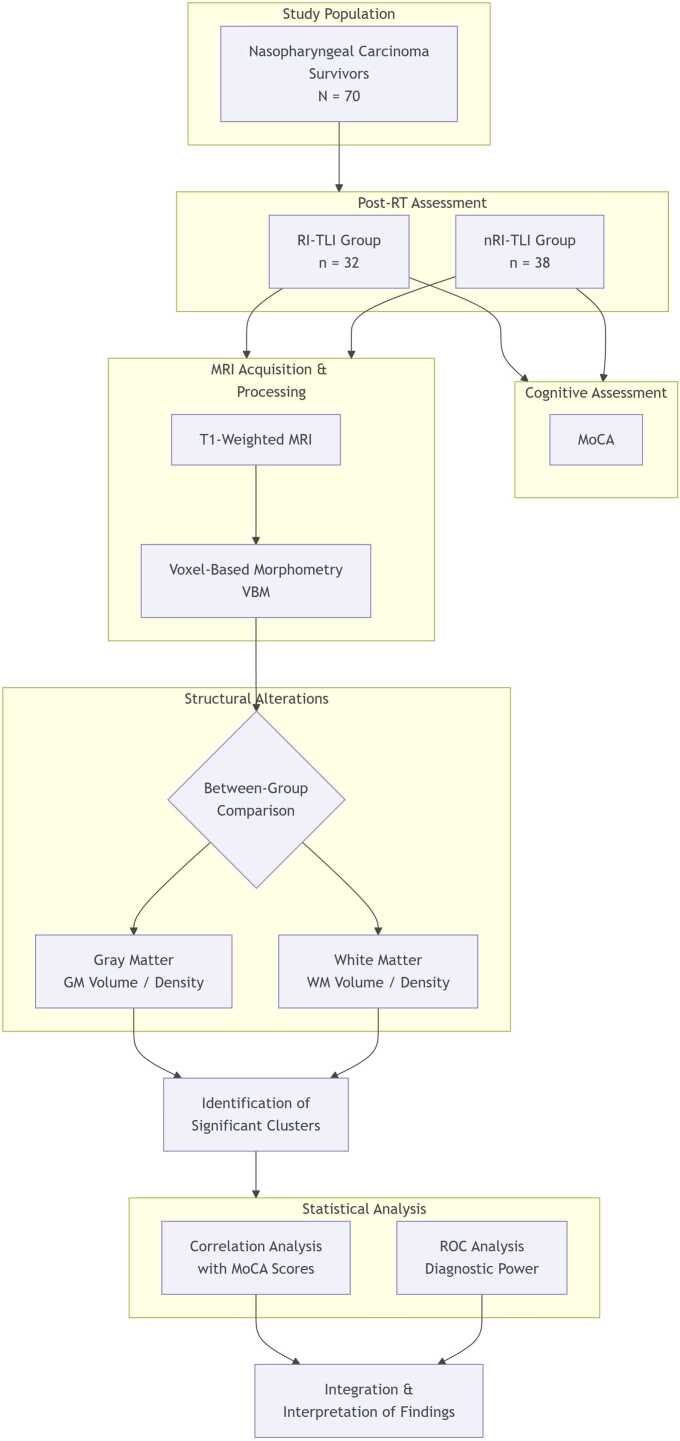
Flowchart of the study design and analytical pipeline. This flowchart delineated the study design for investigating structural brain alterations and cognitive impairment in nasopharyngeal carcinoma (NPC) survivors with and without radiation-induced temporal lobe injury (RI-TLI). A total of 70 NPC survivors were allocated into two groups one-year post-radiotherapy (Post-RT): the RI-TLI group (n = 32) and the non-RI-TLI (nRI-TLI) group (n = 38). All participants underwent concurrent cognitive assessment using the Montreal Cognitive Assessment (MoCA) and T1-weighted structural magnetic resonance imaging (MRI). The MRI data were processed using whole-brain voxel-based morphometry (VBM) to quantify gray matter (GM) and white matter (WM) volume and density. Statistical comparisons between groups identified significant structural alterations, the clusters of which were subsequently correlated with MoCA scores. Finally, receiver operating characteristic (ROC) analysis was performed to evaluate the diagnostic utility of the identified structural biomarkers for distinguishing RI-TLI patients.

This study was approved by the Ethical Commission of Jiangsu Cancer Hospital & Jiangsu Institute of Cancer Research & The Affiliated Cancer Hospital of Nanjing Medical University. All patients signed informed consents before entrance to the study. In addition, all methods were performed in accordance with the Declaration of Helsinki in this study. The study flowchart was shown in Fig. 1.

In this study, NPC was diagnosed by nasopharyngeal biopsy and histopathology according to the 8th edition of AJCC Head and Neck Tumor Staging Criteria for NPC (Huang and O'sullivan, 2017). All patients were pathologically confirmed non-cornification undifferentiated NPC patients. According to the National Comprehensive Cancer Network (NCCN) guidelines for NPC (Zhou et al., 2020b), all patients were irradiated with 28–33 fractions (once a day, quintic a week). The prescribed radiation dose to gross tumor volume of the nasopharynx (GTVnx) was 70–72 Gy, to gross tumor volume of lymph nodes (GTVnd) was 66–70 Gy, to high-risk clinical target volume (CTV1) was 60 Gy, and to low-risk clinical target volume (CTV2) was 50.4 Gy.

We employed the Chinese version of MoCA as a screening tool for cognitive function. The MoCA is a widely used screening scale that can assess multiple cognitive domains, including visuospatial/executive functions, naming, attention, language, abstraction, delayed recall, and orientation. However, it is important to note that, as a screening tool, the precision of its individual sub-scores and its sensitivity to subtle changes in specific cognitive domains are limited. Therefore, the analysis results for specific cognitive domains (e.g., attention, delayed recall) in this study should be considered preliminary indications based on a screening tool, rather than definitive diagnoses of specific cognitive impairments.

NPC patients treated with the above treatment plan in our institution were reviewed at the time of one year after the end of radiotherapy. Patients who met the following criteria were included in this study: (1) Han Chinese ethnicity; (2) right-handed, (3) aged from 20 to 60 years old; (4) more than 9 years of education. Additionally, all patients reported no cognitive impairment before radiotherapy, and their MoCA total scores were all ≥ 26. Patients who combined with other malignant tumors were excluded. All patients had no history of clinically diagnosed psychiatric disorders (e.g., major depression, anxiety disorders, schizophrenia) at enrollment and had not used medications known to significantly affect cognitive function within one month prior to assessment. During the radiotherapy period, no patients used medications widely recognized to impact cognitive function. Finally, a total of 32 NPC patients with RI-TLI and 38 NPC patients without RI-TLI matched in terms of gender, age and education level, were included in this study. The MoCA was applied to evaluate the cognitive function of all patients on the day of structural MRI scan. MoCA was used to evaluate different cognitive domains, including visual space and executive function, naming, attention, language, abstract understanding, delayed recall and orientation. To assess intergroup comparability and control for potential confounding factors, we collected demographic data (gender, age, years of education) and key radiotherapy dose parameters for all patients.

The sample size (n = 70) was determined based on feasibility and is comparable to previous VBM studies investigating structural changes in NPC patients’ post-radiotherapy. A post-hoc power analysis using G*Power indicated that with n = 70, α= 0.05 (two-tailed), and assuming a medium effect size (f²=0.15), the study achieved a power of > 0.80 to detect significant group differences in a two-group design.

### 2.2 MRI data acquisition

MRI data were acquired with a 3.0 T Philips Aachieva Scanner. Structural MRI data were acquired using a T1-weighted 3D turbo spin echo (3D TSE) sequence with following parameters: repetition time (TR)= 500ms; echo time (TE)= 12ms; flip angle (FA)= 90°; matrix= 340 × 216; voxel= 0.68 mm× 0.85 mm× 5 mm; slice thickness= 5 mm; interval= 1 mm; number of slices= 36.

All structural MRI data were visually inspected post-acquisition to exclude images with obvious motion artifacts or distortions. To ensure the quality of VBM analysis, we further utilized the quality assessment module within the DPARSF toolkit to check image contrast, signal-to-noise ratio, and motion parameters. All included data met the pre-set quality standards.

It is acknowledged that the T1-weighted sequence used in this study employed a slice thickness of 5 mm, which does not represent the optimal isotropic acquisition typically recommended for VBM analysis. This parameter choice may introduce partial volume effects and pose challenges for precise gray-white matter segmentation and spatial normalization. However, given the clinical and logistical constraints of the study, this was the available standardized structural protocol. To mitigate the potential impact, we employed the DARTEL algorithm for normalization, which is designed to handle inter-subject anatomical variations more robustly than standard approaches, and applied smoothing (FWHM=4 mm) which is a standard step in VBM to reduce noise and residual misalignment. The results should be interpreted with this methodological consideration in mind.

### 2.3 MRI data analysis

MRI data were preprocessed using the software of DPARSF (Chao-Gan and Yu-Feng, 2010). The detailed steps of voxel-based morphometry (VBM) analysis were as follows: (1) reorientation of T1-weighted images; (2) segmenting each image into gray matter, white matter and cerebrospinal fluid; (3) normalization using the DARTEL; (4) modulating the normalized images with the Jacobian determinants; (5) smoothing with a Gaussian kernel of 4-mm full width at half maximum. The modulated, normalized and smoothed images of volume and density of GM (GM volume and GM density) and WM (WM volume and WM density) were used for the statistical analysis.

### 2.4 Statistical analysis

The MoCA scores were compared between RI-TLI and nRI-TLI groups. The voxel-wise differences of volume and density of GM and WM between RI-TLI and nRI-TLI groups were compared by two-sample *t*-tests using the software of Resting-State fMRI Data Analysis (REST) (Song et al., 2011). The statistical threshold for these group comparisons was set at a voxel-level *P* < 0.001, corrected for multiple comparisons using the AlphaSim method (cluster size > 50 voxels). Following the identification of significant clusters, the mean values of GM/WM (volume and density) were extracted from each cluster and defined as regions of interest (ROIs). The relationships between these ROI-based GM/WM measures and MoCA scores were then explored across all participants using *Pearson* correlation analysis. Given the exploratory nature of analyzing correlations across multiple ROIs and cognitive domains, the significance level for these correlations was controlled for multiple comparisons using the False Discovery Rate (FDR) procedure, with a threshold of q< 0.05. Effect sizes for correlations were interpreted based on Cohen’s guidelines (small: *r* = 0.1, medium: *r* = 0.3, large: *r* = 0.5). Finally, ROC curve was performed to determine the suitability of altered brain structure for distinguishing RI-TLI from nRI-TLI. The ROC analysis aimed to evaluate the combined discriminatory efficacy of GM/WM volume and density metrics from brain regions showing significant between-group differences. Specifically, the metric values across all significant ROIs for each subject were integrated into a composite score via a logistic regression model. This composite score was then used to generate the ROC curve. *P* < 0.05 was considered to be statistically significant difference.

## 3 Results

### 3.1 Demographic and clinical characters between RI-TLI and nRI-TLI groups

Table 1 compares the demographic and clinical characteristics between the RI-TLI and nRI-TLI groups. The results show no statistically significant differences between the two groups in key demographic variables, including gender distribution, age, and years of education. Regarding the core radiotherapy dose parameters, the prescribed doses to the gross tumor volume of the nasopharynx (GTVnx) and the gross tumor volume of lymph nodes (GTVnd) were also highly similar between the two groups, with no significant differences. This provides support for the comparability of the two groups in terms of demographic baseline and main treatment intensity.

**Table 1:** Comparison of demographic and clinical characters between RI-TLI and nRI-TLI groups.

	**RI-TLI (n = 32)**	**nRI-TLI (n = 38)**	***χ***^***2***^**/*****t***	***P***
**Gender (male/female)**	19/13	21/17	0.12	0.73
**Age (years)**	53.00 ± 8.50	54.16 ± 8.04	-0.58	0.56
**Educational level (years)**	12.47 ± 2.79	13.08 ± 2.59	-1.02	0.31
**Radiotherapy regimen (Gy)**				
GTVnx	71.19 ± 0.74	71.21 ± 0.74	-0.13	0.90
GTVnd	68.28 ± 1.35	67.95 ± 1.51	0.97	0.34
CTV1	60	60	-	-
CTV2	50.4	50.4	-	-
Left temporal lobe Dmax	70.20 ± 4.96	69.31 ± 5.28	0.68	0.50
Right temporal lobe Dmax	70.16 ± 5.35	70.87 ± 4.59	-0.56	0.58
Left hippocampal Dmax	47.49 ± 14.18	47.36 ± 14.54	0.04	0.97
Right hippocampal Dmax	48.92 ± 15.13	49.46 ± 14.08	-0.15	0.88

GTVnx: radiation dose to gross tumor volume of the nasopharynx; GTVnd: radiation dose to gross tumor volume of lymph nodes; CTV1: radiation dose to high-risk clinical target volume; CTV2: radiation dose to low-risk clinical target volume.

### 3.2 Difference of MoCA scores between RI-TLI and nRI-TLI groups before and after radiotherapy

Table 2 presents the comparison of MoCA scores between the two groups before radiotherapy and one year after radiotherapy. Before radiotherapy, there were no statistically significant differences between the two groups in either the MoCA total score or any of the cognitive domain sub-scores (all *P* > 0.05), indicating comparable baseline cognitive function. At one year post-radiotherapy, the RI-TLI group showed significantly lower scores than the nRI-TLI group in attention, delayed recall, and the MoCA total score (all *P* < 0.01). No significant differences were found between the two groups in the sub-scores for visuospatial/executive functions, naming, language, abstraction, and orientation. These results indicate that patients with RI-TLI exhibit more pronounced overall cognitive decline, and the screening results suggest they may have relatively prominent difficulties in attention and delayed memory.

**Table 2:** Comparison of MoCA scores between RI-TLI and nRI-TLI groups before and after radiotherapy.

	**RI-TLI (n = 32)**	**nRI-TLI (n = 38)**	***t***	***P***
**MoCA before radiation**				
Visual space and executive function	4.47 ± 0.51	4.55 ± 0.50	0.69	0.49
Naming	2.59 ± 0.50	2.63 ± 0.49	0.32	0.75
Attention	5.41 ± 0.50	5.53 ± 0.51	1.00	0.32
Language	2.75 ± 0.44	2.63 ± 0.49	-1.06	0.29
Abstract understanding	1.47 ± 0.51	1.40 ± 0.50	-0.62	0.54
Delayed recall	4.63 ± 0.49	4.58 ± 0.50	-0.39	0.70
Orientation	6.00 ± 0.00	6.00 ± 0.00	-	-
Total scores after radiation	27.31 ± 1.06	27.32 ± 0.84	0.01	0.99
**MoCA after radiation**				
Visual space and executive function	4.34 ± 0.48	4.47 ± 0.51	-1.09	0.28
Naming	2.44 ± 0.50	2.40 ± 0.50	0.36	0.72
Attention	4.47 ± 1.05	5.24 ± 0.85	-3.39	< 0.01
Language	1.88 ± 0.79	2.00 ± 0.77	-0.67	0.51
Abstract understanding	1.59 ± 0.50	1.50 ± 0.51	0.78	0.44
Delayed recall	3.56 ± 1.22	4.24 ± 0.88	-2.68	< 0.01
Orientation	6.00 ± 0.00	6.00 ± 0.00	-	-
**Total scores after radiation**	24.28 ± 1.73	25.84 ± 1.84	-3.64	< 0.01

RI-TLI: radiation-induced temporal lobe injury; nRI-TLI: without radiation-induced temporal lobe injury; MoCA: Montreal Cognitive Assessment. *P* values were obtained by two sample *t*-tests and the statistical significance level was set at *P* < 0.05.

### 3.3 Difference of GM between RI-TLI and nRI-TLI groups

**Fig. 2:**
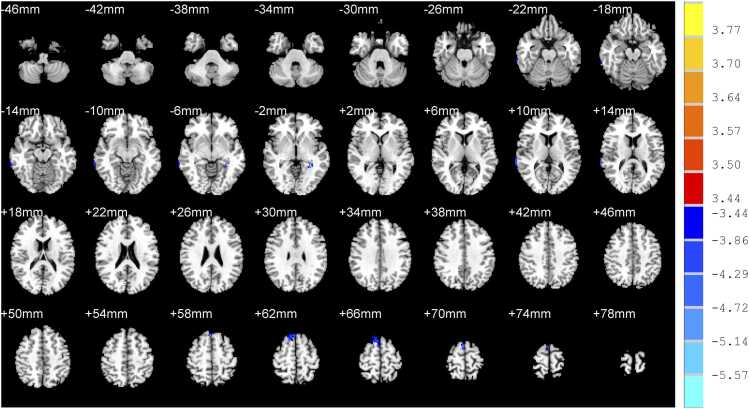
Difference of volume of grey matter between RI-TLI and nRI-TLI groups. RI-TLI: radiation-induced temporal lobe injury; nRI-TLI: without radiation-induced temporal lobe injury. Compared with nRI-TLI group, RI-TLI group exhibited decreased GM volume in the right middle temporal gyrus, left hippocampus, right superior temporal gyrus, right superior frontal gyrus.

**Fig. 3:**
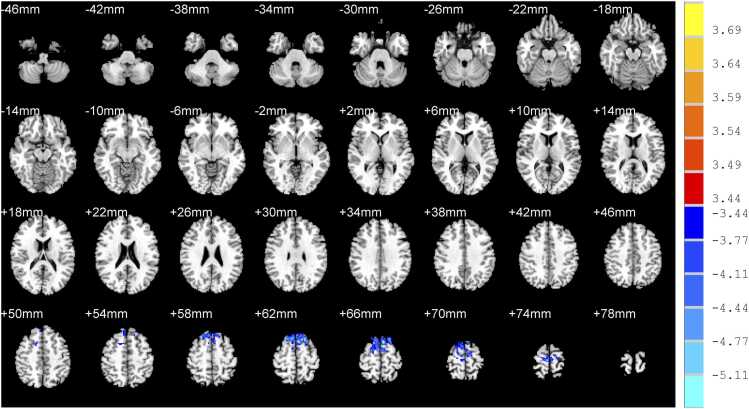
Difference of density of grey matter between RI-TLI and nRI-TLI groups. RI-TLI: radiation-induced temporal lobe injury; nRI-TLI: without radiation-induced temporal lobe injury. Compared with nRI-TLI group, RI-TLI group exhibited decreased GM density in the left superior frontal gyrus, right superior frontal gyrus, right supplementary motor area.

Compared with nRI-TLI group, RI-TLI group exhibited decreased GM volume in the right middle temporal gyrus, left hippocampus, right superior temporal gyrus, right superior frontal gyrus and decreased GM density in the left superior frontal gyrus, right superior frontal gyrus, right supplementary motor area. (Table 3; Figs. 2&3)

**Table 3:** Difference of GM between RI-TLI and nRI-TLI groups.

	**Peak MNI coordinates**			**Clusters**	**Peak*****T*****values**
	x	y	z		
**GM volume**					
Right middle temporal gyrus	72	-36	-8	74	-4.10
Left hippocampus	-32	-42	-2	57	-5.99
Right superior temporal gyrus	70	-38	14	60	-4.85
Right superior frontal gyrus	4	6	72	234	-4.99
**GM density**					
Left superior frontal gyrus	-20	22	64	461	-5.44
Right superior frontal gyrus	12	20	64	838	-4.99
Right supplementary motor area	4	-16	72	122	-4.97

RI-TLI: radiation-induced temporal lobe injury; nRI-TLI: without radiation-induced temporal lobe injury; GM: grey matter. MNI: montreal neurological institute. Peak *T* values were obtained by two sample *t*-tests and corrected by the AlphaSim program in the REST software (*P* < 0.001, cluster size > 50 voxels).

### 3.4 Difference of WM between RI-TLI and nRI-TLI groups

**Fig. 4:**
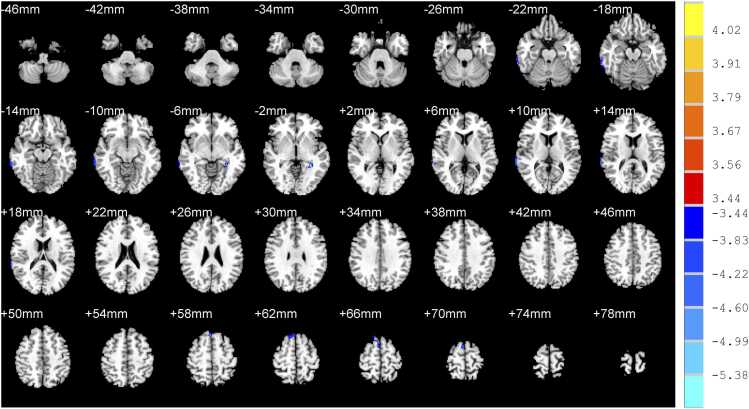
Difference of volume of white matter between RI-TLI and nRI-TLI groups. RI-TLI: radiation-induced temporal lobe injury; nRI-TLI: without radiation-induced temporal lobe injury. Compared with nRI-TLI group, RI-TLI group demonstrated decreased WM volume in the right inferior temporal gyrus, left hippocampus, right superior temporal gyrus, right medial superior frontal gyrus.

**Fig. 5:**
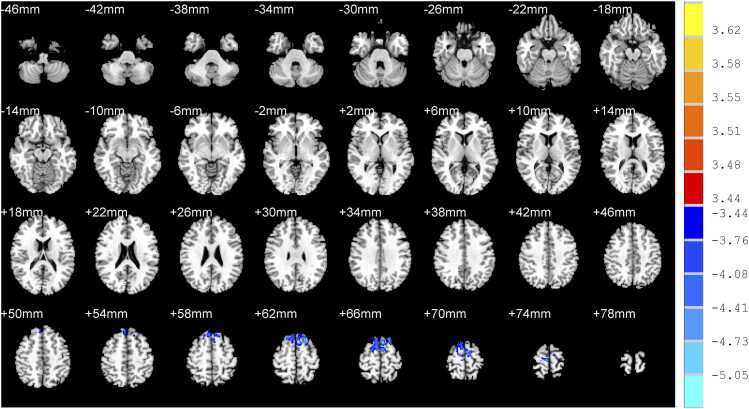
Difference of density of white matter between RI-TLI and nRI-TLI groups. RI-TLI: radiation-induced temporal lobe injury; nRI-TLI: without radiation-induced temporal lobe injury. Compared with nRI-TLI group, RI-TLI group demonstrated decreased WM density in the left superior frontal gyrus, right superior frontal gyrus.

In addition, RI-TLI group demonstrated decreased WM volume in the right inferior temporal gyrus, left hippocampus, right superior temporal gyrus, right medial superior frontal gyrus and decreased WM density in the left superior frontal gyrus, right superior frontal gyrus. (Table 4; Figs. 4&5)

**Table 4:** Difference of WM between RI-TLI and nRI-TLI groups.

	**Peak MNI coordinates**			**Clusters**	**Peak*****T*****values**
	x	y	z		
**WM volume**					
Right inferior temporal gyrus	68	-40	-16	138	-4.26
Left hippocampus	-32	-42	-2	60	-5.77
Right superior temporal gyrus	70	-36	14	80	-5.13
Right medial superior frontal gyrus	4	30	58	136	-4.73
**WM density**					
Left superior frontal gyrus	-6	16	62	496	-5.37
Right superior frontal gyrus	20	22	62	495	-4.64

RI-TLI: radiation-induced temporal lobe injury; nRI-TLI: without radiation-induced temporal lobe injury; WM white matter. MNI: montreal neurological institute. Peak *T* values were obtained by two sample *t*-tests and corrected by the AlphaSim program in the REST software (*P* < 0.001, cluster size > 50 voxels).

### 3.5 Relationships between GM and WM and MoCA scores

**Fig. 6:**
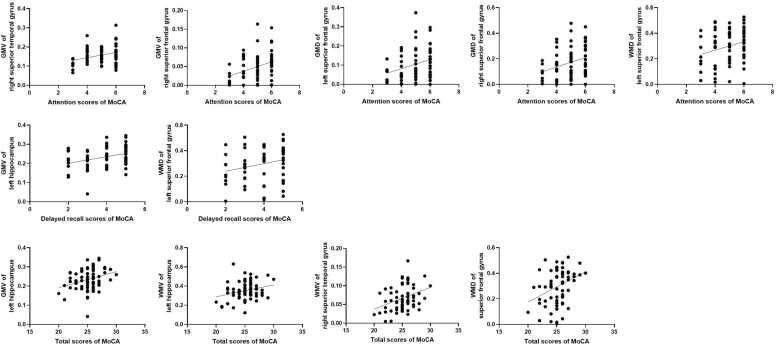
Relationships between GM and WM and MoCA scores. GM: grey matter; WM: white matter; MoCA: Montreal Cognitive Assessment.

The attention scores of MoCA were positively associated with GM volume of the right superior temporal gyrus (*r* = 0.31; *P* = 0.01), right superior frontal gyrus (*r* = 0.35; *P* < 0.01), GM density of the left superior frontal gyrus (*r* = 0.28; *P* = 0.02), right superior frontal gyrus (*r* = 0.32; *P* < 0.01), WM density of the left superior frontal gyrus (*r* = 0.24; *P* = 0.04). In addition, the delayed recall scores of MoCA were positively related to GM volume of the left hippocampus (*r* = 0.35; *P* < 0.01), WM density of the left superior frontal gyrus (*r* = 0.24; *P* = 0.04). Moreover, the total scores of MoCA were positively associated with GM volume of the left hippocampus (*r* = 0.31; *P* = 0.01), WM volume of the left hippocampus (*r* = 0.26; *P* = 0.03), right superior temporal gyrus (*r* = 0.35; *P* < 0.01), WM density of the left superior frontal gyrus (*r* = 0.34; *P* < 0.01). All represented small-to-medium effect sizes according to Cohen’s criteria. (Fig. 6)

Following FDR correction, the correlation of attention scores of MoCA and GM volume of right superior frontal gyrus remained statistically significant. In addition, the correlation of delayed recall scores of MoCA and GM volume of left hippocampus remained statistically significant. Moreover, the correlations of total scores of MoCA and WM volume of right superior temporal gyrus, WM density of right superior frontal gyrus.

### 3.6 The value of altered brain structure for distinguishing RI-TLI from nRI-TLI

**Fig. 7:**
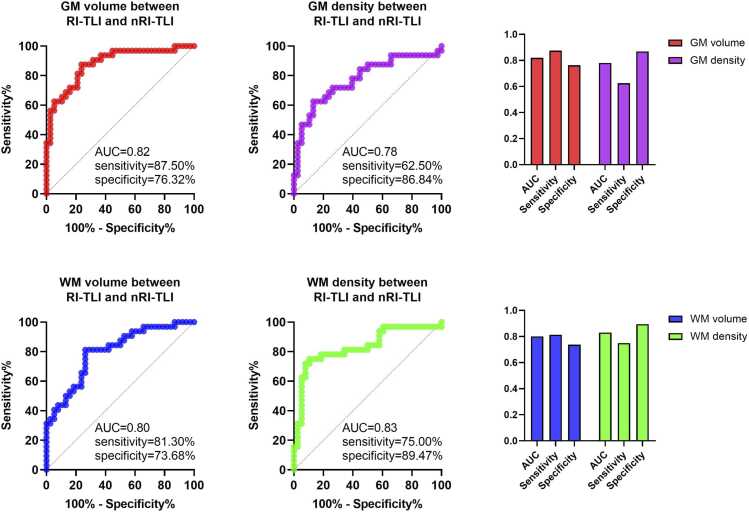
The value of altered brain structure for distinguishing RI-TLI from nRI-TLI. RI-TLI: radiation-induced temporal lobe injury; nRI-TLI: without radiation-induced temporal lobe injury.

ROC analysis revealed that altered brain structure might be helpful for distinguishing RI-TLI from nRI-TLI (GM volume: sensitivity=87.50%, specificity=76.32%, AUC=0.88, CI: 0.80–0.96; GM density: sensitivity=62.50%, specificity=86.84%, AUC=0.78, CI: 0.66–0.89; WM volume: sensitivity=81.30%, specificity=73.68%, AUC=0.80, CI: 0.70–0.90; WM density: sensitivity=75.00%, specificity=89.47%, AUC=0.83, CI: 0.73–0.93) (Fig. 7).

## 4 Discussion

In this study, one year after the end of radiotherapy, NPC patients with RI-TLI showed decreased MoCA scores when compared with those without RI-TLI. In addition, RI-TLI group exhibited decreased GM and WM in both the temporal lobe and other regions across the whole brain when compared with nRI-TLI group. Moreover, more declined MoCA scores in RI-TLI group were related to the decreased GM and WM of brain regions outside the temporal lobe. ROC analysis demonstrated that altered GM and WM might be helpful for distinguishing RI-TLI from nRI-TLI. In this cross-sectional study, we found that NPC patients with RI-TLI one year post-radiotherapy showed more severe cognitive impairment and more extensive reductions in GM and WM structure compared to those without RI-TLI. The co-occurrence of these structural alterations with cognitive deficits suggests a possible link between the presence of RI-TLI and a broader pattern of brain structural compromise that extends beyond the temporal lobe.

A strength of this study is our confirmation that the two groups had no significant differences in cognitive function before radiotherapy. This, to some extent, reduces the interference of pre-existing individual cognitive differences on the outcomes and strengthens the plausibility that the observed post-radiotherapy cognitive and structural differences may be attributable to radiotherapy and its related complications (e.g., RI-TLI). In addition, in this study, we compared key demographic variables (such as age and education level) and treatment variables (such as radiation doses to key targets) that could potentially influence cognitive and brain structural outcomes between the groups. The results showed no significant differences in these potential confounders between the groups, strengthening the inference that the observed intergroup differences (in cognitive scores, gray matter/white matter volume/density) are more likely to be related to the RI-TLI status itself or its underlying biological processes, rather than being driven by differences in these fundamental variables.

In NPC patients who have received radiotherapy, the decline in neurocognitive ability may be attributed to brain injury induced by radiation (Makale et al., 2017). The structural markers of brain impairments and cognitive dysfunction appeared after radiotherapy (Chapman et al., 2012). In NPC patients, radiation therapy can affect normal brain tissue and sometimes lead to severe delayed sequelae, known as radiation-induced encephalopathy. It is usually manifested as serious cognitive problems, which seriously affect the survival and quality of life of NPC patients. Using the MoCA screening, this study found that patients with RI-TLI demonstrated more significant overall cognitive decline, and the screening results suggested their attention and delayed recall domains might be relatively more affected. However, we must interpret these findings cautiously. As an important limitation of this study, the MoCA is primarily a screening tool; its sub-tests lack the depth and complexity required to precisely define or quantify the degree of impairment in specific cognitive domains. Therefore, the observed differences in 'attention' and 'delayed recall' scores are more appropriately interpreted as indications of relative weakness in these domains during screening. Future research needs to employ more comprehensive, standardized neuropsychological test batteries to validate and precisely characterize the profile of cognitive impairment associated with RI-TLI.

Although WM demyelination is considered as the histological presentation of radiation injury in the brain (Zhou et al., 2017), the pathogenesis of RI-TLI is still difficult to fully understand. Radiation encephalopathy is considered a disease primarily limited to the temporal lobe, and structural and functional abnormalities in brain regions outside of the temporal lobe are largely overlooked. Therefore, conducting whole brain assessment on NPC patients receiving radiotherapy is crucial for better understanding the neural mechanisms of RI-TLI, and this is possible with the advancement of MRI.

Altered whole-brain functional connectivity was found in NPC patients who had received radiotherapy when compared with those who had not received radiotherapy (Ma et al., 2016, Ma et al., 2017). In addition, increased brain activity in the inferior temporal lobe at the early-delayed stage could be predict the development of brain damages in the future (Ding et al., 2018). In this study, the results indicate that, compared to the nRI-TLI group, patients with RI-TLI exhibit significant reductions in both the volume and density of grey matter and white matter across multiple key brain regions. These differences are primarily concentrated in the temporal lobe (particularly the right middle temporal gyrus, right superior temporal gyrus, right inferior temporal gyrus, and left hippocampus) and the prefrontal lobe (bilateral superior frontal gyrus and supplementary motor area). This finding partially aligns with the traditional view that radiation encephalopathy primarily affects the temporal lobe (Dong et al., 2024), while simultaneously revealing a more extensive whole-brain involvement pattern, especially notable in the significant impairment of frontal lobe structures (Brummelman et al., 2012). Given that RI-TLI may be irreversible and incurable (Ding et al., 2018, Lin et al., 2017), exploring the structural and functional differences in the whole brain between patients with RI-TLI and those without RI-TLI is of crucial clinical significance and may help in early identification and timely prevention of RI-TLI.

However, previous studies mainly focused on NPC patients without RI-TLI, with much less attention paid to structural differences in the whole brain between patients with RI-TLI and those without RI-TLI after radiotherapy. In this study, from a functional perspective, these affected brain regions are core hubs that constitute advanced cognitive (Millan et al., 2016, Berron et al., 2020) and memory (Lee et al., 2022, Rolls, 2022) networks. The hippocampus is a critical structure for the formation of declarative memory (Slotnick, 2024), and its damage is directly associated with the memory impairments (Krishnan et al., 2006) commonly observed in RI-TLI patients. The superior and middle temporal gyri are involved in auditory processing (Zaykova et al., 2025), language comprehension (Battistella et al., 2020), and semantic memory (Noulhiane et al., 2007). Abnormalities in these areas may explain the declines in verbal fluency or comprehension observed in some patients. More importantly, this study identified extensive structural damage in the frontal lobe system (including the superior frontal gyrus and supplementary motor area). The frontal cortex is crucial for executive functions (such as working memory, cognitive flexibility, planning, and decision-making) (Tandetnik et al., 2021), attention regulation (Boroujeni et al., 2025), and motor planning (Rolls et al., 2023). Damage to these regions may underlie the reduced executive function, slow information processing speed, and diminished behavioral motivation seen in RI-TLI patients, significantly impacting their daily life and quality of living.

In this study, abnormalities in white matter structure (such as the reduced white matter density in the frontal and temporal lobes) may reflect radiation-induced axonal damage or demyelination. This could lead to decreased efficiency of information transmission between various functional networks in the brain, exacerbating diffuse cognitive impairments (Hilal et al., 2021). The whole-brain assessment results from this study suggest that the neuropathological mechanisms of RI-TLI are not limited to focal lesions in the temporal lobe but involve a distributed brain network impairment that includes the temporo-frontal system (Kourtidou et al., 2022). This structural disruption at the network level may be the fundamental cause of its complex and persistent cognitive sequelae (Ma et al., 2022). Future research could combine functional magnetic resonance imaging to further investigate how these structural changes lead to functional connectivity disruptions in specific brain networks (such as the default mode network (Yang et al., 2023) and the frontoparietal control network (Chen et al., 2022)). This would provide more precise imaging biomarkers for the early identification of high-risk RI-TLI patients and the development of targeted neuroprotective intervention strategies.

Furthermore, following FDR correction, the correlations between attention scores and GM volume of the right superior frontal gyrus, delayed recall scores and GM volume of the left hippocampus, as well as total MoCA scores and WM volume of the right superior temporal gyrus and WM density of the right superior frontal gyrus remained statistically significant. These results indicate that, although some associations weakened after correction, the structural integrity of brain regions such as the right superior frontal gyrus, left hippocampus, and right superior temporal gyrus—particularly their gray matter volume and white matter structure closely related to advanced cognitive networks—remains stably associated with specific cognitive impairments in NPC patients following RI-TLI. Notably, the critical role of the right superior frontal gyrus in attention and overall cognitive function, as well as the central position of the hippocampus in memory encoding and retrieval, have been supported by numerous functional imaging studies [references (Chua et al., 2016; Chang et al., 2021)]. This study further provides direct structural evidence for the correlation between these brain regions and cognitive impairment, suggesting that RI-TLI may lead to declines in cognitive functions such as attention and memory by affecting the structural integrity of these core brain regions. These associations that remain significant after correction particularly highlight the potential central role of the right fronto-temporal network in the cognitive sequelae related to RI-TLI, warranting further attention in future longitudinal and mechanistic studies.

ROC analysis demonstrated that brain structural metrics based on gray matter volume, gray matter density, white matter volume, and white matter density all exhibited good discriminatory efficacy in distinguishing RI-TLI from nRI-TLI patients. Notably, the composite score for gray matter volume achieved the highest AUC (0.88), indicating that significant gray matter atrophy plays a central role in this differentiation. It is particularly noteworthy that the gray matter volume metric maintained high sensitivity (87.50%) while also achieving a specificity of 76.32%, suggesting its ability to effectively identify RI-TLI patients while reliably excluding non-RI-TLI individuals. The white matter density metric, on the other hand, demonstrated the highest specificity (89.47%), implying greater reliability in identifying non-RI-TLI individuals. These findings collectively indicate that RI-TLI patients exhibit a characteristic pattern of brain structural alterations, which not only correlates statistically with cognitive impairment but also holds potential for translation into clinically practical diagnostic indicators. Future studies could employ methods such as machine learning to further integrate multimodal imaging and clinical features, thereby constructing more accurate predictive models to provide objective imaging evidence for the early identification and intervention of RI-TLI.

There are no statistically significant differences between the RI-TLI and nRI-TLI groups in the maximum radiation doses delivered to the temporal lobes or hippocampi. This result significantly enhances the interpretability and scientific value of our study. Rather than simply confirming a dose-response relationship, our results point to a more complex and interesting phenomenon. The fact that patients who developed RI-TLI received statistically equivalent maximum doses to these critical structures, compared to those who did not, suggests that the observed structural and cognitive differences cannot be attributed solely to the prescribed dose or the peak point dose. This shifts the focus toward other critical factors, such as: (1) Individual Radiosensitivity: There may be intrinsic biological (e.g., genetic or molecular) differences that predispose certain patients to more extensive normal tissue damage following radiation. (2) Dose Distribution Effects: While the Dmax is similar, the volume of tissue receiving high doses, the specific location of the dose "hot spots," or the dose to specific subregions (e.g., the perirhinal cortex vs. the primary auditory cortex) may differ between groups and be more predictive of injury. (3) Pre-existing Vulnerability: Unmeasured factors such as microvascular health, inflammatory status, or baseline white matter integrity could modulate the brain's response to an equivalent radiation insult. Our findings provide strong evidence that the presence of RI-TLI is a marker of, or contributor to, a broader, diffuse process of radiation-induced brain injury affecting distributed cognitive networks, and that this vulnerability exists independently of the maximum point dose to the temporal lobes.

However, several limitations should be mentioned in this study. Firstly, although we have conducted balance tests for key demographic and treatment dose variables between the groups, there may still be other unmeasured potential confounding factors, such as subtle vascular risk factors, genetic susceptibility, specific medication use during treatment (e.g., steroids, chemotherapeutic agents), and psychosocial factors, all of which could potentially influence cognitive outcomes or brain structure. Future studies should systematically collect and control for these variables to more precisely elucidate the independent association between RI-TLI and brain structural alterations. Secondly, and most critically, this study has a cross-sectional design. Although we confirmed comparable cognitive baselines before radiotherapy, this cannot substitute for longitudinal tracking data to establish causality. We observed intergroup 'state' differences at the single time point of 'one year post-radiotherapy,' not differences in the 'process of change.' Therefore, it is impossible to definitively conclude whether RI-TLI causes widespread gray and white matter structural alterations throughout the brain, or whether some common predisposing factor or a more extensive radiation damage process both leads to RI-TLI and causes these brain structural changes. Future prospective longitudinal studies incorporating neuroimaging at baseline before radiotherapy and at multiple time points after radiotherapy are needed to reveal the precise temporal sequence and causal mechanisms. Thirdly, and critically, the structural MRI acquisition parameters, particularly the 5-mm slice thickness, were suboptimal for VBM analysis. While we utilized advanced processing tools (e.g., DARTEL) and standard smoothing to enhance robustness, the non-isotropic voxels inherently increase the risk of partial volume effects and may compromise the precision of tissue segmentation and spatial normalization. This represents a significant methodological constraint that could affect the sensitivity and specificity of the detected volumetric and density differences. Therefore, the reported findings, while internally consistent and correlated with behavior, should be viewed as preliminary and require replication with high-resolution, isotropic 3D T1-weighted sequences in future studies. Finally, there are limitations regarding the cognitive assessment tool used in this study. Although the MoCA is an efficient and convenient screening tool and has proven useful in identifying mild cognitive impairment, its sub-tests are insufficient for detecting subtle changes in specific cognitive domains (e.g., executive function, processing speed, working memory) or for precise grading of impairment severity. Relying on MoCA sub-scores to infer the degree of impairment in specific cognitive domains may be an oversimplification. Therefore, our conclusions regarding impairments in specific domains such as attention and memory are preliminary and suggestive, requiring confirmation through more comprehensive, multi-dimensional neuropsychological assessments.

## 5 Conclusion

In summary, our findings demonstrate that NPC patients who developed RI-TLI exhibit more pronounced cognitive deficits and more widespread GM/WM structural alterations than those who did not, and that the severity of cognitive impairment is correlated with the degree of structural change in specific brain regions. While our cross-sectional design cannot establish causality, these associations raise the possibility that RI-TLI is a marker of, or contributes to, a more diffuse process of radiation-induced brain injury affecting cognitive networks. The identified structural alterations showed promising discriminatory value, suggesting their potential utility as neuroimaging biomarkers for RI-TLI.

## CRediT authorship contribution statement

**Yizhi Ge:** Writing – review & editing, Writing – original draft, Visualization, Validation, Supervision, Software, Resources, Project administration, Methodology, Investigation, Formal analysis, Data curation, Conceptualization. **Jianfeng Wu:** Resources, Project administration, Methodology, Investigation, Formal analysis, Data curation, Conceptualization. **Pengwei Yan:** Methodology, Investigation, Formal analysis, Data curation. **Jingjing Han:** Writing – review & editing, Writing – original draft, Visualization, Validation, Supervision, Resources, Project administration, Methodology, Investigation, Formal analysis, Data curation, Conceptualization. **Yesong Guo:** Writing – review & editing, Writing – original draft, Visualization, Validation, Supervision, Software, Resources, Project administration, Methodology, Investigation, Formal analysis, Data curation, Conceptualization. **Siwen Liu:** Writing – review & editing, Writing – original draft, Visualization, Validation, Supervision, Software, Resources, Project administration, Methodology, Investigation, Funding acquisition, Formal analysis, Data curation, Conceptualization. **Peng Xie:** Writing – review & editing, Writing – original draft, Visualization, Validation, Supervision, Software, Resources, Project administration, Methodology, Investigation, Formal analysis, Data curation, Conceptualization.

## Compliance with ethical standards

This study was approved by the Ethical Commission of Jiangsu Cancer Hospital & Jiangsu Institute of Cancer Research & The Affiliated Cancer Hospital of Nanjing Medical University. All patients signed informed consents before entrance to the study. In addition, all methods were performed in accordance with the Declaration of Helsinki in this study.

## Funding

The work was supported by the grants of: Jiangsu Province Young Scientific and Technological Talents Support Project (No. JSTJ-2025-401).

## Declaration of Competing Interest

We declare that we have no conflict of interest.

## Data Availability

The data of this study are available from the corresponding author upon request.
